# Fundamentals of Bowel Cancer for Biomedical Engineers

**DOI:** 10.1007/s10439-023-03155-8

**Published:** 2023-02-14

**Authors:** Jiyuan Tian, Kenneth Omokhagbo Afebu, Andrew Bickerdike, Yang Liu, Shyam Prasad, Bradley J. Nelson

**Affiliations:** 1 Engineering Department, University of Exeter, North Park Road, Exeter, EX4 4QF UK; 2 Royal Devon University Healthcare NHS Foundation Trust, Barrack Road, Exeter, EX2 5DW UK; 3 Multi-Scale Robotics Lab, ETH Zürich, Tannenstrasse 3, 8092 Zurich, Switzerland

**Keywords:** Bowel cancer, Bowel cancer biomechanics, Bowel cancer diagnosis, Bowel cancer metastasis, Colorectal cancer

## Abstract

Bowel cancer is a multifactorial disease arising from a combination of genetic predisposition and environmental factors. Detection of bowel cancer and its precursor lesions is predominantly performed by either visual inspection of the colonic mucosa during endoscopy or cross-sectional imaging. Most cases are diagnosed when the cancer is already at an advanced stage. These modalities are less reliable for detecting lesions at the earliest stages, when they are typically small or flat. Removal of lesions at the earliest possible stage reduces the risk of cancer death, which is largely due to a reduced risk of subsequent metastasis. In this review, we summarised the origin of bowel cancer and the mechanism of its metastasis. In particular, we reviewed a broad spectrum of literatures covering the biomechanics of bowel cancer and its measurement techniques that are pertinent to the successful development of a bowel cancer diagnostic device. We also reviewed existing bowel cancer diagnostic techniques that are available for clinical use. Finally, we outlined current clinical needs and highlighted the potential roles of medical robotics on early bowel cancer diagnosis.

## Introduction

According to Digestive Cancers Europe,^27^ bowel cancer (specifically cancer of the colon or rectum, collectively known as colorectal cancer) is the second deadliest cancer, responsible for approximately 170,000 deaths every year in the European Union alone. Survival rates vary across European countries due to national variations in healthcare and locally provided treatment strategies.^98^ Although early cancer detection can significantly improve patients’ outcomes, most cases are detected at a late stage once local and distant spread (metastasis) has occurred and when the chance of cure is small. If more cases were diagnosed at an early stage (from the current 13% to a targeted 50%), up to 130,000 more lives could be saved each year and more than €3 billion in healthcare spending could potentially be saved.^27^ In the UK, over 50% of bowel cancer cases are diagnosed at a late stage, and, even prior to the Covid-19 pandemic, 20–64% of National Health Service (NHS) endoscopy units in the four nations of the UK were failing to meet urgent suspected cancer targets.^116^ Shortages of endoscopists and nursing staff were cited as the highest barriers to meeting demand. Thus, in order to improve survival rates and reduce the heavy burden of bowel cancer on patients, healthcare systems, and the wider economy, more effective screening techniques and programs for early cancer detection must be developed and introduced.

Detection of bowel cancer and its precursor lesions (dysplastic polyps) is currently performed by either visual inspection of the colonic mucosa during endoscopy or cross-sectional imaging. Most cases are diagnosed when the cancer is already at an advanced stage. According to van Cutsem* et al*.,^138^ approximately 25% of patients present with metastases (distantly spread disease) at initial diagnosis, and almost 50% of patients with bowel cancer will develop metastases during the course of their disease, contributing to their poor outcomes. However, these diagnostic modalities are less reliable for detecting lesions at the earliest stages, when they are typically small or flat. Removal of lesions at the earliest possible stage reduces the risk of subsequent cancer death. This is largely due to a reduced risk of subsequent metastasis. A further limitation of endoscopy has been the burden placed on patients, clinicians and healthcare systems by the rapid expansion of its use in recent years. Some patients experience significant pain during procedures, which require a team of clinicians to sedate and monitor them, while maintaining and decontaminating the increasingly complex and expensive devices. For clinicians to acquire safely the required expertise and practice, lengthy training periods (typically 2–5 years) and highly developed professional regulatory frameworks are required. Furthermore, despite major advances in image acquisition and processing over recent decades, the basic design and ergonomics of endoscopes have barely changed in more than 40 years. Therefore, in gastrointestinal endoscopic practice, there is an urgent need for new modalities that are safe, painless, reliable, cost-effective and efficient, and which require minimal training for practitioners.

**Figure 1:**
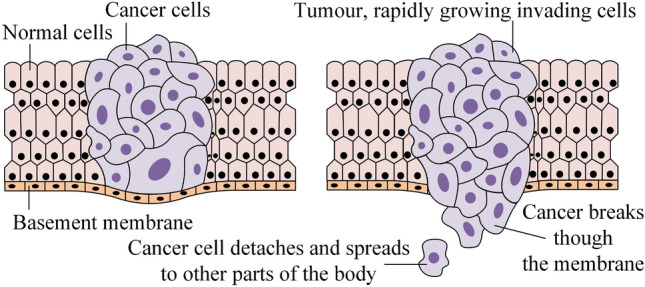
As tumours grow and cancer cells accumulate more and more genetic changes, they grow through the bowel wall and become metastatic.^13^ Some cells acquire the ability to move from the primary tumour into the bloodstream or lymphatic system to migrate to a distant location in the body, and to grow into tumours in the new location. These locations are typically distant lymph nodes or other organs, such as the liver and lungs.

As stated above, metastasis accounts for most cancer-related deaths, largely because metastatic cancer is notoriously challenging to treat. Most cancer researchers have assumed that the metastasis of tumours typically occurs later in the disease process. The general idea has been that as tumours grow and cancer cells accumulate more and more genetic changes, some cells acquire the ability to move from the primary tumour into the bloodstream or lymphatic system, as illustrated in Fig. 1, to migrate to a distant location in the body, and to grow into tumours in the new location.^13^ These locations are typically distant lymph nodes or other organs, such as the liver and lungs. Large metastases can be detected by cross-sectional imaging (typically computed tomography, magnetic resonance imaging or positron-emission tomography scans). However, early metastases are often too small to be detected by these modalities, and current blood tests perform poorly in this regard. The assessment of the metastatic potential of a localised tumour and the prediction of future metastasis is extremely important in clinical practice, both for prognosis and for choice of therapy. At present, this assessment is incomplete and relies on (i) the estimated stage of the lesion detected at presentation, (ii) the detection of malignant cells in draining lymph nodes following scans or surgery, (iii) the detection of malignant cells in blood vessels or lymphatics within a resected tumour, (iv) the histological type of tumour within a biopsy or resection specimen, (v) the detection of particular genetic mutations or changes within a tumour specimen, and (vi) clinical judgement. Furthermore, recent findings^1,20,44^ showed that many bowel cancers could have spread from the site where they first formed to other parts of the body (so-called micrometastasis) long before the original tumour can even be detected by current screening tests. There is, therefore, a pressing need to develop new techniques that can detect early metastasis or the metastatic potential of tumours at presentation and before treatment decisions are made.

This paper will review the fundamentals of bowel cancer, including its occurrence, mechanism of metastasis, biomechanical characteristics and existing diagnostic techniques. The rest of the paper is organised as follows. In “Occurrence of Bowel Cancer” section, we summarise the occurrence of bowel cancer, including its classification and pathogenesis. In “Mechanism of Metastasis” section, the mechanism of metastasis is discussed. In “Biomechanics of Bowel Cancer” section, we discuss the biomechanics of bowel cancer and, in particular, review different methods of measuring the stiffness of bowel cancer tissues. In “Existing Diagnostic Techniques” section, we briefly review some of existing techniques used for bowel cancer diagnosis and staging. Finally, conclusions and future outlook are drawn in “Conclusions and Future Outlook” section.

## Occurrence of Bowel Cancer

Bowel cancer is a multifactorial disease that combines two conventional disciplines, epidemiology and pathology, utilising different approaches to understand the disease. This multidisciplinary investigation includes an understanding of different aspects involved in the carcinogenesis process, such as endogenous (e.g., genetic) components and exogenous (e.g., microenvironment variation and lifestyle) components.^3^ The majority of bowel cancer cases are sporadic, caused by environmental factors rather than a hereditary predisposition or family history.^3,132^ The minority of cases that have either a family history or a clear inheritance indicate the presence of inherited genetic mutations in addition to precipitant environmental factors.^132,150^

### Classification of Bowel Cancer

Bowel cancer can be classified epidemiologically into three types: sporadic, familial and hereditary.^4,114^ The majority of bowel cancer, between 60 and 80%, is sporadic and appears to be unrelated to any familial or inherited predisposition. It is more common in the people over the age of 50, most likely as a result of the effects of ageing and longer exposure to dietary and environmental factors in causing the acquired genetic mutations that drive carcinogenesis. The familial type accounts for 20–40% of the cases but is not associated with specific, inherited, single-gene mutations. Population studies demonstrate that there is a high risk of developing this tumour when family members of primary consanguinity have sporadic bowel cancer, and the risk is two to three times higher than the general population. The hereditary type makes up perhaps 5% of cases and comprises several distinct clinical and pathological entities that can be distinguished both by specific single-gene mutations transmitted within families and by tumour pathology. Such cancers may emerge* via* one of several increasingly well-described pathways characterised by intermediate steps associated with acquired genetic changes, such as adenomatous polyps, flat adenomas and sessile serrated adenomas.^4^

### Pathogenesis of Bowel Cancer

As a heterogeneous disease, bowel cancers have different mechanisms of pathogenesis.^132,150^ The adenoma-carcinoma sequence was reported in 1980, elucidating the metamorphosis of normal colorectal epithelium to adenoma, and then to invasive and metastatic tumours.^4,77^ Advances in molecular biology and cancer genetics over the last two decades have further enhanced our understanding of the pathogenesis of both sporadic, familial and hereditary bowel cancer syndromes.^132^ More recently, changes in the intestinal microenvironment caused by variations in the microbiome have been proposed to form one link between environmental agents and tumour development at the cellular level, although potential specific mechanisms underlying these processes are only now beginning to be elucidated.

**Figure 2:**
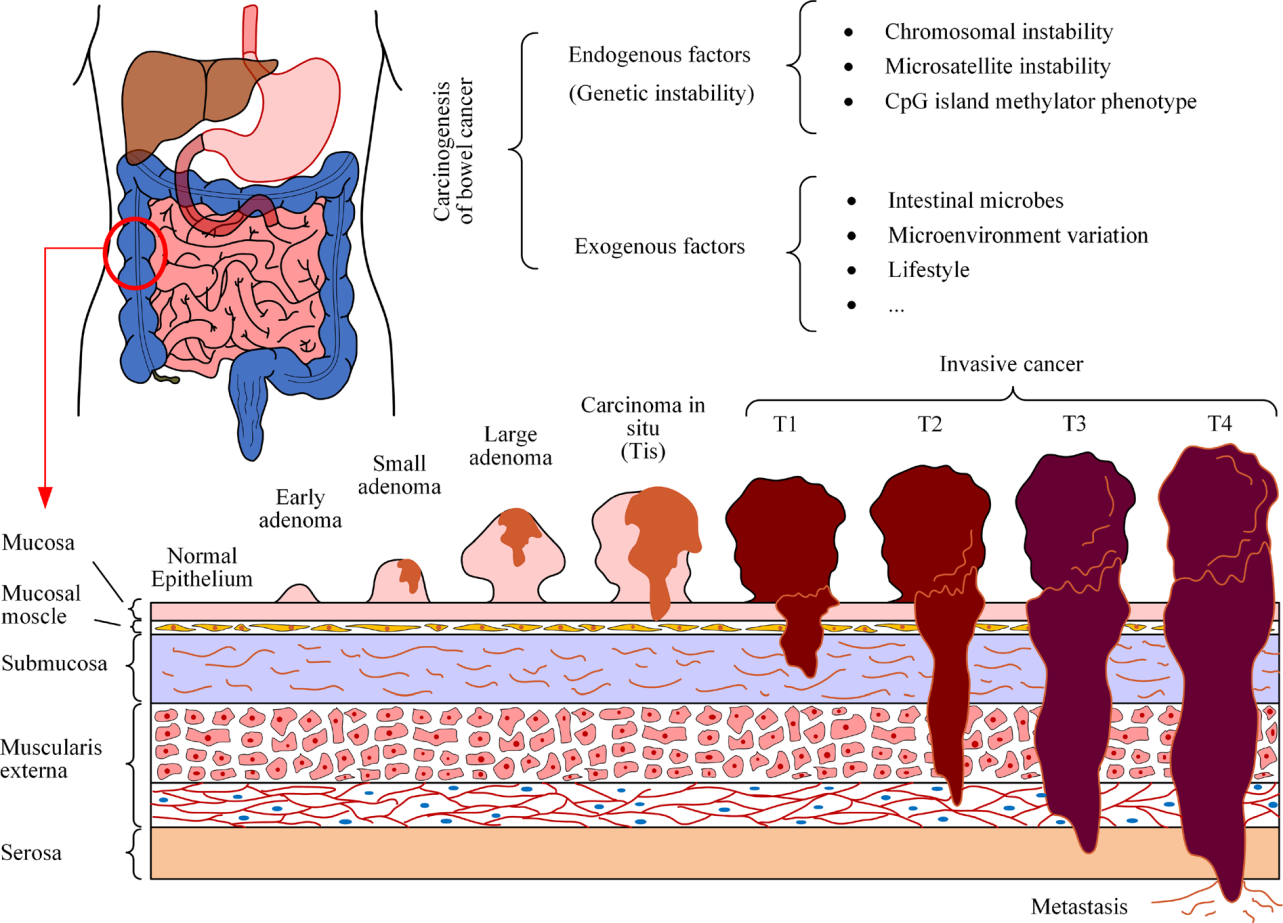
Schematic depiction of adenoma-carcinoma progression sequence and subsequent primary tumour T stages. Multidisciplinary study of carcinogenic factors involving endogenous and exogenous components in colon cancer pathogenesis is summarised. The adenoma-carcinoma sequence links carcinogenic factors and the approximate order of morphological changes that appear at different stages of tumour development, starting with steps that promote benign tumour formation, followed by steps that promote progression toward histologically more advanced tumours, and finally steps associated with tumour transformation into an invasive carcinoma, including stages T1, T2, T3 and T4.

The accumulation of acquired genetic and epigenetic alterations that turn normal glandular epithelial cells into invasive adenocarcinomas is a critical element of bowel cancer development. The polyp-to-cancer progression sequence, also known as the adenoma-carcinoma sequence, was postulated in Vogelstein and Fearon’s pioneering and classic tumour development model.^140^ It connects genetic alterations to the order in which tumour morphology alters at different stages of cancer development, beginning with a step that promotes the formation of benign neoplasms (e.g. adenomas and sessile serrated polyps), then a step that promotes the progression to more histologically advanced neoplasms, and finally a step that transforms the tumours to invasive carcinoma,^33,36^ see Fig. 2. It is worth noting that most bowel cancers are thought to develop* via* the adenoma-carcinoma sequence and that, in most cases, the adenomatous stage is associated with polyp formation.^50^ Typically, one or more adenomas will develop during the human lifetime, but the vast majority of benign adenomas do not invade the mucosa and become malignant carcinomas. In addition, the duration of adenoma-carcinoma sequences is often estimated to average more than 10 years, making it difficult to observe* in vivo*.^65^ Once the tumour has become invasive, it can penetrate the colonic lining and invade nearby structures (Fig. 2). The tumour’s stage at diagnosis is, in fact, a strong determinant of the overall prognosis and outcome. In terms of the most commonly used TNM staging system for bowel cancer, T staging is as follows: Tis (tumour in situ) means the cancer is only growing into the mucosa but no further; T1 tumours have invaded deep to the mucosa into the next layer of the bowel wall (submucosa); T2 tumours have grown into the muscle layer (muscularis); T3 tumours have invaded through the muscularis into the subserosa but not through the membrane covering the outside of the bowel; a T4 staging indicates that the tumour has extended beyond the outer wall of the colon and has invaded the adjacent structures.^50^ In addition, lymphatic, haematogenous, and peritoneal spread may also occur, known as metastasis and are detailed in the N and M components of TNM staging.

Aside the original and well-established linear adenoma-carcinoma sequence model of bowel cancer, carcinogenesis is also thought to be more heterogeneous and characterised by at least three distinct pathways: ‘conventional’ (adenoma-carcinoma sequence), ‘alternative’, and ‘serrated’. In brief, the ‘conventional’ route is assumed to entail specific genetic and chromosomal alterations, including mutations in the APC (Adenomatous Polyposis Coli) gene and loss of heterozygosity, which form part of the chromosomal instability pathway that leads to cancer.^36^ The ‘alternative’ method might include KRAS gene and APC mutations. Although alternative paths may be more diverse and have fewer traits, ‘conventional’ and ‘serrated’ pathways appear to be more homogenous and distinct. The ‘serrated’ route originated from alterations in CpG methylation and typically involves BRAF gene mutations and the late development of microsatellite instability.^36^ Therefore, the morphological changes of the adenoma-carcinoma sequence, various gene mutations and genetic instability always develop in parallel.

The genomic instability of bowel cancer and its pathogenesis are mediated by three primary pathways: chromosomal instability, microsatellite instability, and CpG island methylator phenotype. Bowel cancer develops from one or more of these factors, resulting in deregulation of various signalling pathways, thereby leading to deregulation of cell proliferation, apoptosis and differentiation.^36,77,132^ The chromosomal instability pathway is an adenoma-carcinoma sequence pathway in which mutational activation of oncogenes or tumour suppressor inactivation leads to malignant transformation. The microsatellite instability pathway is characterised by a clonal increase in the number of repeated DNA nucleotide units in microsatellites, resulting from the inactivation of mismatch repair (MMR) genes. The pathway of CpG island methylator phenotype is described by the global hypermethylation of promoter CpG island sites, which also results in the inactivation of tumour suppressor genes.^3,130^ However, these pathways do not occur in isolation but overlap to some extent.^36^ In the conceptualisation of these pathways, although there are still some uncertainties in the molecular mechanisms underlying the development of colorectal tumours from early lesions to advanced ones, most bowel cancers develop slowly from adenomatous polyps or flat adenomas.^3,132^

Interestingly, bowel cancers that occur on the left side (descending colon, sigmoid colon and rectum) and right side (transverse colon, ascending colon and caecum) have different biological and clinical characteristics. Right-sided bowel cancer is more likely to have genome-wide hypermethylation of CpG island methylator phenotype, leading to poor prognostic characteristics.^132^ In addition, right-sided cancers are often exogenous, that is, the tumour grows outward from a location in the intestinal wall. Left-sided cancers are often endogenous and encircling, thus obstructing the intestines.^114,132^

Epidemiological and cross-sectional studies led to the proposal that the gut microbiome is closely associated with the development of bowel cancer.^107,118,157^ The intestinal bacterial flora that make up this microbiome achieve carcinogenic effects by destroying the homeostasis of the microenvironment, changing the immune response and the biofilm of intestinal bacteria, and producing toxic metabolites.^118,157^ In detail, intestinal microbes can induce abnormal immune responses in colonic tissues, weakening the intestinal epithelial barrier, and producing tumorigenic toxins that can act on intestinal epithelial cells and cause cell proliferation, thereby forming a special immune microenvironment. In addition, these toxins produce carcinogenic metabolites, reactive oxygen species, and other free radicals, which can cause DNA damage in host cells and induce mutations.^107^ These mechanisms are held to jointly promote the development of colorectal tumours. It has been found that the presence of some bacteria is closely related to bowel cancer, but their carcinogenic effects have only been confirmed in animal experiments. Clinical evidence is limited to the increase in the positive rate or proportion of these bacteria in the intestinal flora of bowel cancer patients. It is not clear whether these bacteria cause tumours or whether changes in the intestinal microenvironment are beneficial to their growth. Epidemiological evidence is still very limited, hence, further research is needed to reveal the complex relationship between gut microbes and bowel cancer.^118,132^

In addition to the above carcinogenic mechanisms, transcription factors are also the main driving factors for colorectal tumours. They are often dysregulated in bowel cancer due to gene amplification, mutation, genetic instability, epigenetic or post-transcriptional modification, resulting in altered gene expression and cellular function.^150^ The risk of developing bowel cancer is also clearly associated with exogenous factors, such as lifestyle, environmental and socioeconomic factors. Malignant changes in some colon cells may be the result of long-term exposure to carcinogens. Thus, a combination of acquired genetic mutations due to environmental exposure to carcinogens and, in some, inherited genetic predisposition appears to provoke the development of adenomas and cancers. Dietary factors, obesity, smoking and heavy alcohol consumption are probably the most common risk factors associated with bowel cancer incidence.^3^ However, understanding all the mechanisms and associations between these factors is a complex area of study and is still in progress.

## Mechanism of Metastasis

Cancer metastasis is the process whereby primary tumour cells spread from the original tumour site and establish secondary tumour colonies in distant organs. Complex, and made up of multiple stages, the metastatic cascade includes angiogenesis, epithelial mesenchymal transition, tumour cell invasion into the surrounding tissue matrix, tumour cell intravasation into the circulatory system, survival in the blood or lymphatic vessels, tumour cell extravasation into the distant organ, proliferation and colonisation of the newly established secondary tumour at the new site.

In 2019, NHS data showed that metastasis was present in 19.4% of patients in England at initial diagnosis of bowel cancer.^89^ Prognosis for patients with metastatic bowel cancer is poor, with only 10% of diagnosed Stage IV patients surviving for five years or more after initial diagnosis.^86^ The most common site of metastasis from bowel cancer is the liver, and this is often the only site where metastasis is observed. It is thought that this is due to the direct draining of the colon’s blood supply to the liver* via* the portal venous circulation.^112^ It is therefore imperative when engineering robotic devices for diagnosis to understand the mechanisms involved in the spread of cancer cells from the primary tumour site to distant organs and the potential difficulties encountered.

### Angiogenesis and the Tumour Microenvironment

Once a primary bowel tumour reaches a volume of approximately 2 mm^3^, it relies on the formation of new vascular networks to provide a suitable supply of nutrients and oxygen and facilitate the removal of waste. The processes in which new blood and lymphatic vessels form is called angiogenesis and lymphangiogenesis, respectively.^95^ The newly formed angiogenic tumour capillaries resemble a chaotic network of structurally weak, leaky and inconsistent blood vessels. Blood flow within the tumour blood vessels can also be intermittent and bi-directional. The combined mechanical forces within the tumour microenvironment are primarily made up of solid and fluid stresses. Solid stress is the combination of mechanical forces exerted by the growing tumour and the resistance to deformation of the surrounding tissue.^96^ As the tumour tissue becomes stiffer than the surrounding stromal tissue, solid stresses accumulate, and the surrounding stromal tissue is deformed. Collagen fibres that make up the extracellular membrane assist the transmission of these forces through the tumour and to the surrounding tissue. Characteristically, collagen fibres stiffen in tension and provide tensile strength and further resistance to stretching of the tissue surrounding the tumour. Within the tumour microenvironment, fluid stress is the accumulation of interstitial fluid pressure, microvascular fluid pressure and fluid shear stress exerted by the blood flow. Interstitial fluid pressure is increased throughout tumour proliferation due to vascular leaking and reduced lymphatic draining. The heightened interstitial fluid pressure within the tumour creates a pressure gradient against the normal interstitial pressure of the adjacent tissue and can encourage cell escape from the primary tumour.^53^ As a result, both solid and fluid stresses pose two obvious limitations when considering the efficacy of therapeutic treatment. First, compressive stress on the blood vessels leading to collapse or deformation impedes or prevents the effectiveness of treatments that utilise the circulatory system. Second, an elevated level of interstitial pressure reduces the effectiveness of perfusion of administered drugs inside the tumour.

### Epithelial-Mesenchymal Transition and Extracellular Membrane Stiffening

To gain access to the blood stream, cancer cells must first detach from the tumour and invade through the extracellular matrix or infiltrate local tissue. Epithelial mesenchymal transition is the process in which tumour associated epithelial cells change phenotype and transform to acquire more invasive and metastatic mesenchymal cell properties.^55^ In contrast to the tightly packed epithelial cells, held together by junction molecules, the mesenchymal cells possess no cell adhesion molecules and therefore acquire a higher capacity for migration.^133^ In bowel cancer, it has been observed that the stiffness of tumour increases with the progression of cancer.^57^ The increase in tumour stiffness has also been determined to activate growth factors known to promote epithelial mesenchymal transition in tumours situated in the colon.^6^ Though the stiffness of the tumour microenvironment increases in response to tumour growth, the compliance of cancer cells has been shown to increase. Through micropipette aspiration experimentation, Stage IV (SW48) bowel cancer cells were shown to be more deformable than primary Stage I (HT29) bowel cancer cells, with mean instantaneous and equilibrium Young’s modulus values calculated as 574.72 and 84.76 Pa for Stage IV and 331.27 and 123.47 Pa for Stage I bowel cancer cells, respectively.^102^ Modification to the organisation within the cell cytoskeleton leads to increased cell deformability, therefore aiding the metastatic capabilities of the cancer cells to survive the mechanical stress they experience throughout the metastatic cascade.

### Invasion and Intravasation

The tumour microenvironment is made up of inflammatory and immune cells, including cancer associated fibroblasts, neutrophils and macrophages, and environmental conditions, such as signalling molecules and the extracellular membrane. Local invasion of the tumour cells starts with the degradation of the basement membrane, followed by the migration of tumour cells through the surrounding stroma towards nearby tissue or neighbouring blood and lymphatic vessels, highly facilitated by angiogenesis. Tumour cells invade and navigate through surrounding tissue until they encounter nearby blood or lymphatic vessels. Upon encountering the vessels, the tumour cells enter circulatory systems through a process called intravasation. Intravasation is a notoriously inefficient process,^25^ with the occurrence more common in the blood stream than in the lymphatic and is an essential stage in the metastatic cascade.

### Survival in the Blood Stream

When primary tumour cells successfully migrate into the blood stream through intravasation, they become circulating tumour cells (CTCs) and are considered as one of the main biomarkers for metastatic spread. In the blood, the occurrence of these cells is low in percentage, with the number of CTCs found in patients with metastatic cancer in the region of 1–10 CTCs out of $10^9$ blood cells.^83^ Furthermore, the percentage of CTCs that successfully survive to form metastatic colonies is as low as 0.02%.^39^ They circulate either as individual cells or less commonly bunched together in clusters. To survive in the blood stream, CTCs resist destructive mechanisms, including programmed cell death after detachment from the primary tumour and haemodynamic shear forces. Within the circulation, these cells have been shown to bind to platelets, offering shielding from the mechanical stress of blood, inhibiting apoptosis and mediating the process of CTC extravasation.^94^ Increased collisions between platelets and CTCs have also been shown to increase the blood-borne metastasis of bowel cancer.^79^ The main hydrodynamic force CTCs may experience is fluid shear stress, caused by the flow of blood across the cells’ surface and influenced by both the fluid velocity and viscosity. Mean levels of fluid shear stress range from 1–2, 0.1−0.4 and 0.4–3 Pa for capillaries, veins and arteries, respectively.^34^ Colorectal CTCs have been recorded to exhibit resistance to fluid shear stress. CTC adhesion to the endothelial wall is necessary for extravasation. However, the high shear stress CTCs experience within the vasculature negatively affects the ability they have to form stable cell-cell adhesions. Hence, extravasation is often observed at locations of vascular bifurcation or due to the entrapment of CTCs in the narrow capillaries.

### Tumour Cell Extravasation and Metastatic Colonisation

For the tumour cells to successfully proliferate and start secondary tumours in distant sites, CTCs must first exit the blood stream* via* the process of extravasation. Extravasation starts with the initial arrest of the CTCs to the endothelial cells that make up the walls of the blood vessels caused by the binding between adhesion molecules and the CTC^37^ or by physical entrapment due to the diameter of the CTC being larger than that of the blood capillaries. Tumour cells then squeeze through the endothelial cell junctions or in rarer cases directly penetrate through individual endothelial cells into the surrounding tissue of the new organ. Extravasated tumour cells must then survive in the new tissue to form micrometastasis which often stay dormant for an unknown period before favourable environmental conditions trigger proliferation into macro metastatic tumours. The most common site for CRC metastasis formation is the liver, thought to be caused by the draining of the colon by the portal vein.

**Figure 3:**
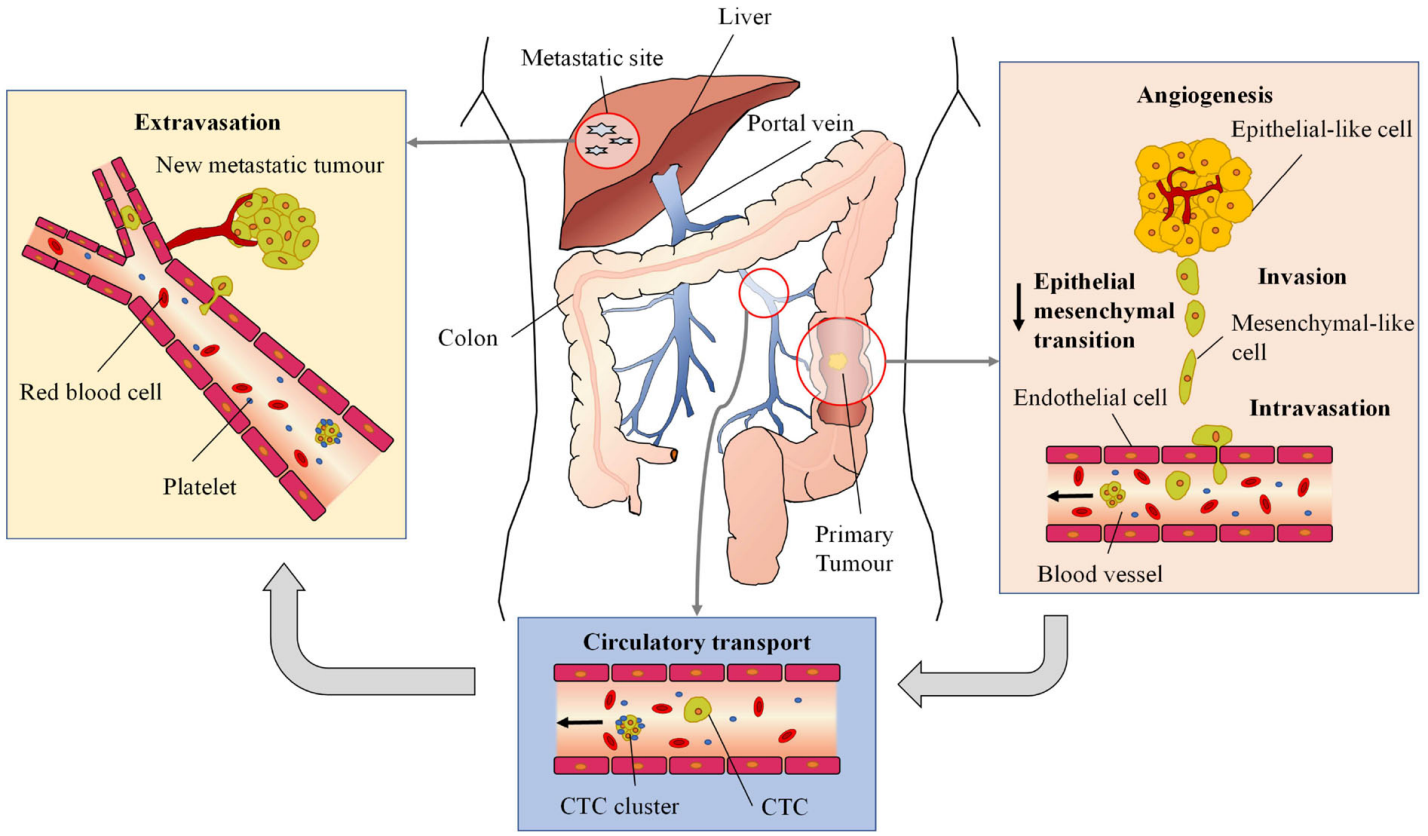
The metastatic colorectal cancer cascade, which describes the series of processes whereby tumour cells migrate to distant organs by exiting the primary tumour site. Firstly, tumour vasculature is established through angiogenesis, and tumour cells undergo EMT and leave the primary site* via* the circulatory system through intravasation. CTCs in the circulatory system experience fluid shear stress and bind to platelets in the blood to survive. Due to physical trapping in the capillaries or by binding to the blood vessel walls, CTCs arrest in the vasculature of the secondary organ and extravasate, finally forming a new metastatic tumour.

In summary, the entire cycle of bowel cancer metastasis is graphically presented in Fig. 3.

## Biomechanics of Bowel Cancer

The development of bowel cancer is a multistep and multifactorial-driven process. In recent years, more attention has been paid not only to the morphological and molecular screening of the tissue samples but also to their mechanical properties, in order to fully understand the physiological and pathological processes at the cell and tissue level.^24^ Every process from bowel cancer cell initiation to cancer cell metastasis manifests mechanical changes at the cellular level, at the level of extracellular matrices and at the tissue level. Cells, as the initial mutated unit, exhibit sclerosis during development, and the degree of sclerosis varies among cell types. Recent findings highlight that physical constraints exerted by the extracellular matrix on the epithelium participate in the regulation of cell phenotypes and behaviours. Extracellular matrix exerts these constraints on the epithelial cells through specific topography, stiffness and deformability. Under pathological contexts, such as chronic inflammation or cancer, mechanical properties of the extracellular matrix are profoundly modified, participating in the development and progression of diseases, such as inflammatory bowel disease or bowel cancer.^100^ In addition, tissue can be modelled as a complex fibrous extracellular matrix and cells within the matrix network. Therefore, the increase in tissue stiffness is manifested microscopically as a combined action of cells and extracellular matrix.^154^ However, the changes in the mechanical properties of the tissue itself will also serve as an important mechanical marker of bowel cancer at the macro level, so it has become a macroscopic indicator for identifying the development stage and metastatic behaviour of cancer to a certain extent. In clinical scenarios, minimally invasive or non-invasive methods to detect the mechanical properties of bowel cancer tissue* in vivo* will serve as an effective way to obtain these mechanical markers, especially through the existing endoscopic biopsy techniques or potential capsule endoscopic approaches. In order to gain a preliminary understanding of mechanical properties of healthy and malignant tissues and the potential cancerous transition, this section will discuss different existing detection methods and summarise relevant findings combined with their corresponding pathological phenomena.

### Fundamentals of Tissue Biomechanics

**Figure 4:**
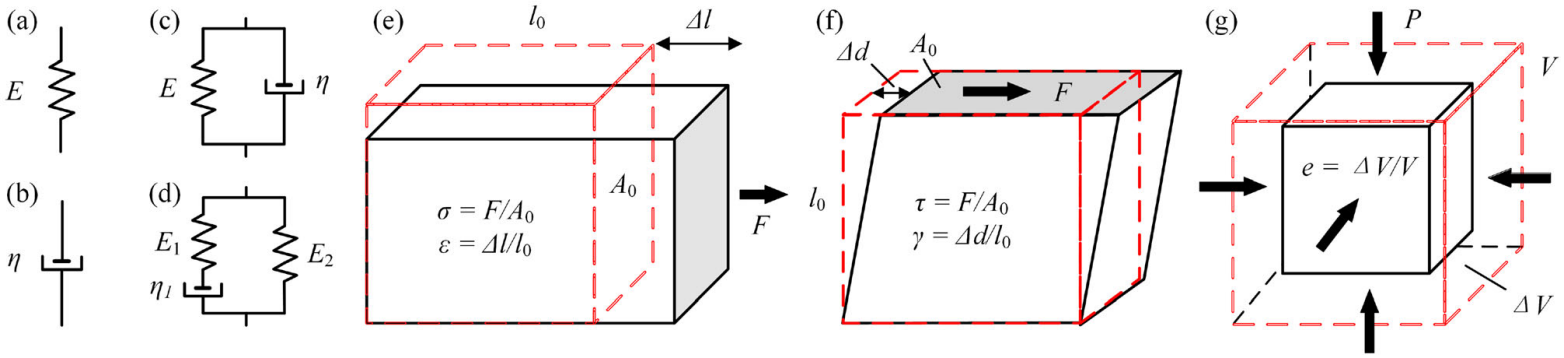
Schematics for depicting the stress–strain relationship of soft tissue: (a) linear elastic model, (b) viscous model, (c) Kelvin–Voigt viscoelastic model and (d) Maxwell viscoelastic model, where *E*, $E_1$ and $E_2$ are the Young’s moduli of the springs and $\eta$ is the viscosity coefficient of the damper. Elasticities of soft tissues are expressed by elastic moduli as (e) Young’s modulus, (f) shear modulus and (g) bulk modulus. In (e), $\sigma$ is the externally applied stress that equals the ratio of applied force *F* to the original applied area $A_{0}$, and $\varepsilon$ denotes for the measured strain that equals the ratio of elongated length $\Delta {l}$ to the original length $l_{0}$. In (f), $\tau$ is the shear stress which equals the ratio of applied force *F* to the area $A_{0}$ on which the force acts, and $\gamma$ denotes for the shear strain that equals the ratio of transverse displacement $\Delta {d}$ to the initial length $l_{0}$. In (g), *P* is the applied pressure, and *e* is the volumetric strain that equals the ratio of altered volume $\Delta {V}$ to the initial volume of the tissue *V*.

Stiffness is resistance to deformation, which can be measured through external forcing. The behaviour exhibited when an external force is applied to the tissue can be described as a viscoelastic body possessing viscosity and elasticity, and it can be approximated by using the models shown in Fig. 4. As can be seen from the physical model in Fig. 4a, the applied stress ($\sigma$) and the measured strain ($\varepsilon$) are proportional, and

1 $$\begin{aligned} E=\frac{\sigma }{\varepsilon } \end{aligned}$$

is the Young’s modulus of the tissue. For the viscosity model illustrated in Fig. 4b, the stress on the damper is proportional to the velocity of deformation, i.e.,

2 $$\begin{aligned} \eta = \sigma /\tfrac{{d}u}{{d}y}, \end{aligned}$$

where $\eta$ is the viscosity coefficient of the damper, *y* is the deformation, *u* is the velocity, and $\tfrac{{d}u}{{d}y}$ is the velocity gradient. Mechanical characteristics of intestine tissue consist of a complex combination of fats, fibres and mucous, showing elastic and viscous behaviours. It is often approximated by using a viscoelastic model, e.g., the Kelvin–Voigt or Maxwell model. The effect of viscosity can be disregarded when the velocity of the external forcing is slow, whereas the viscous component will have the main effect if high frequency vibration is applied and the extent of this influence will depend on the frequency. For the Kelvin–Voigt viscoelastic model shown in Fig. 4c, the stress is governed by

3 $$\begin{aligned} \sigma (t) = E\varepsilon + \eta \tfrac{{d}\sigma (t)}{{d}t}, \end{aligned}$$

where $\tfrac{{d}\sigma (t)}{{d}t}$ is the stress’ rate of change with respect to time, *t*. For the three-element Maxwell viscoelastic model shown in Fig. 4d, the stress–strain relationship can be expressed as

4 $$\begin{aligned} \sigma (t) = \varepsilon \left( E_1 e^{-\frac{E_1}{\eta _1} t } + E_2\right) , \end{aligned}$$

where $E_1$ and $E_2$ are the Young’s moduli of the parallel springs. In addition, the five-element Maxwell model was always used to describe soft tissue, and the number of elements used depends on the tissue’s properties and their experimental testing, e.g., References 59, 104, and 152.

With regard to the elasticity (or stiffness) in statics for a continuous media, it can be quantified by the corresponding moduli, i.e., Young’s modulus (*E*), shear modulus (*G*) and bulk modulus (*K*). They are quantities that measure a material’s resistance to be deformed when uniaxial elongated stress, shear stress or pressure is applied.^93^ According to the Hooke’s law in Eq. (1), as shown in Fig. 4e, the Young’s modulus *E* of a material is defined as the slope of its stress–strain curve in the elastic deformation region, which can be written as

5 $$\begin{aligned} E = \frac{\sigma }{\varepsilon } = \frac{F \cdot {A_{0}}^{-1}}{\Delta {l} \cdot {l_{0}}^{-1}}, \end{aligned}$$

where $\sigma$ is the external applied stress which is equal to the ratio of tension force *F* to original applied area $A_{0}$ and $\varepsilon$ denotes that the measured strain equals the ratio of elongated length $\Delta {l}$ to original length $l_{0}$. One of the applications of Eq. (5) can refer to the design principle of the soft tissue mechanical imaging robot.^23^ Shear modulus *G*, as illustrated in Fig. 4f, can be expressed as

6 $$\begin{aligned} G = \frac{\tau }{\gamma } = \frac{F \cdot {A_{0}}^{-1}}{\Delta {d} \cdot {l_{0}}^{-1}}, \end{aligned}$$

where $\tau$ is the shear stress which is equal to the ratio of applied force *F* to the area $A_{0}$ on which the force acts and $\gamma$ denotes that the shear strain equals the ratio of transverse displacement $\Delta {d}$ to initial length $l_{0}$. The relationship between Young’s modulus *E* and shear modulus *G* can be written as

7 $$\begin{aligned} G = \frac{E}{2(\nu + 1)} \approx \frac{E}{3}, \end{aligned}$$

where the Poisson’s ratio $\nu$ could be set as 0.5 as the content of human tissue is mainly dominated by incompressible water, so *E* will be equal to approximately three times of *G*.^117^ Bulk modulus *K* is defined as the volume changes (volumetric strain *e*) under pressure *P* as shown in Fig. 4g, which can be expressed as

8 $$\begin{aligned} K = \frac{P}{e} = \frac{P}{\Delta {V} \cdot V^{-1}}, \end{aligned}$$

where $\Delta {V}$ is the altered volume and *V* is initial volume of the material. In addition, the Young’s modulus *E* can be expressed as $3K(1-2\nu )$.

### Stiffness of Cancer Tissue

**Figure 5:**
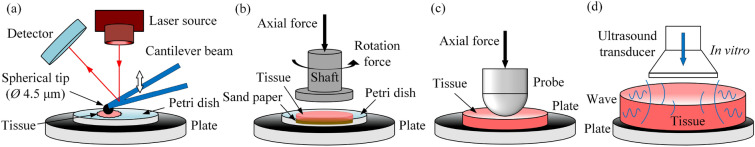
Techniques for measuring the stiffness of cancer tissue: (a) atomic force microscopy (AFM), (b) shear rheometry, (c) tactile sensor and (d) ultrasound elastography (USE). AFM in (a) indents soft biomaterials in force spectroscopy mode to provide nanoscale surface imaging and nanobiomechanical property measurements. The shear rheometry in (b) obtains the stress–strain curves of the material through a controlled cyclical pressure and rotational shear force exerted on the biological tissue sample by a flat probe, so deducing the dynamic elastic modulus and viscoelasticity of the measured tissue. The tactile sensor in (c) measures the force-deformation curve obtained by vertically indenting a rigid spherical indenter probe into the tissue sample and deduces the elastic modulus of the sample under the Hertzian contact theory. USE in (d) is a medical imaging tool that uses low-frequency vibrations during ultrasound to map the elastic properties of soft tissue based on the transmission speed travelling through different tissues.

Fundamentals of biomechanics reviewed above can be applied to study the physicochemical properties of biological materials, such as healthy and malignant tissues. To analyse the mechanical properties of soft biological samples, various techniques, such as atomic force microscopy,^24,115,126^ shear rheometer,^24^ tactile sensor^57,127^ and ultrasound elastography,^73,120,122^ were developed, which are illustrated in Fig. 5. Quick and precise measurements of stiffness and other rheological parameters characterising tissue mechanics, the so-called mechanomarkers, may provide a new means to describe tissue pathology. However, there have been very few reports on the elasticity of gastrointestinal cancer tissue.^57^ Figure 5 shows each experimental development for testing the modulus of elasticity of bowel cancer tissue. In general, existing literatures indicate that bowel cancer tissue is stiffer than healthy tissue. Based on various *in vitro* and *in vivo* tests, the mean values of Young’s moduli of normal bowel tissues are between 0.44 and 9.9 kPa, while those of cancer tissues including all bowel cancer stages measured by different techniques can range from 2.81 to 157.3 kPa.

#### Atomic Force Microscopy

Atomic force microscopy (AFM) provides a method for nanoscale characterisation of a variety of biomaterials, including human tissue. It offers nanoscale surface imaging and nanomechanical characterisation capabilities by indentation of soft biomaterials in a force spectrum mode under physiological conditions.^155^ The main components of AFM shown in Fig. 5a contain a cantilever with a spherical tip (beads with diameters in ranges of approximately 2 to 4.5 $\mu$m), a laser source, a photosensitive photodiode, and a piezoelectric scanner that accurately applies compressive force to soft tissues.^115,126^ Applying a compressive force as a function of the sample’s position in the compression direction may produce a force-distance curve that can be used to calculate the sample’s modulus of elasticity. In the test, the bead-tissue contact area is usually less than 32 $\mu$m^2^, depending on the bead size and depth of indentation. Indentations can be made in multiple locations throughout the central zone of each tumour sample. To determine tissue’s stiffness with a high resolution, colloidal AFM tips can be used as nanoscale indenters in conjunction with Hertz contact mechanics.^24^ The resulting indentation-force curve and geometry properties are then fitted to the Hertz contact model by using

9 $$\begin{aligned}{} & {} E^* = \frac{3}{4} \cdot \frac{F}{\sqrt{R_{\text {e}}} \cdot \sqrt{{\Delta {z}}^3}}, \end{aligned}$$

10 $$\begin{aligned}{} & {} \frac{1}{E^*} = \frac{1 - {{\nu }_{1}}^{2}}{E_1} + \frac{1 - {{\nu }_{2}}^{2}}{E_2} \end{aligned}$$

and

11 $$\begin{aligned} \frac{1}{R_{\text {e}}} = \frac{1}{R_1} + \frac{1}{R_2} \end{aligned}$$

to calculate contact modulus, where $E^*$ is the equivalent Young’s modulus, $E_1$ is the Young’s modulus of the indentor tip, $E_2$ is the Young’s modulus of the tissue sample, *F* is the applied force, $\nu _1$ is the Poisson’s ratio of the indentor tip, $\nu _2$ is the Poisson’s ratio of the tissue sample, $\Delta {z}$ is the mutual approach depth of in the bead-tissue contact point, $R_{\text {e}}$ is the equivalent radius of the two surfaces, $R_1$ is the initial bead radius, and $R_2$ is the initial tissue radius and even can be approximately expressed as the principal radius of curvature of the tissue surface at the origin if the cross-section of the tissue profile is not a circle or a semicircle. However, in cases where the tissue is treated as a flat surface by a rigid spherical tip, the $R_2$ tends to $\infty$, leading to $R_{\text {e}} = R_1$, as well as rigid tip acting on soft tissue makes $E_1 \gg E_2$, the effect of $\frac{1 - {{\nu }_{1}}^{2}}{E_1}$ on $E^*$ will become very small such that it can be ignored, thus Eq. (10) can be simplified as

12 $$\begin{aligned} \frac{1}{E^*} \approx \frac{1 - {{\nu }_{2}}^{2}}{E_2}, \end{aligned}$$

and the Young’s modulus of the tissue sample $E_2$ in the case of considering the measured tissue to be incompressible (${\nu }_{2} \approx 0.5$) can be written as

13 $$\begin{aligned} E_2 \approx \frac{9}{16} \cdot \frac{F}{\sqrt{R_1} \cdot \sqrt{{\Delta {z}}^3}}. \end{aligned}$$

As a result, the mean value of Young’s modulus and the calculated standard deviation for healthy tissue is 0.44 ± 0.3 kPa, whereas for cancerous tissue, it is 5.80 ± 3.8 kPa.^24^ By using the AFM method, the mean values of Young’s moduli for healthy and malignant tissues are significantly different, with the cancerous tissue having a much higher value than the healthy one. Cancer’s development is linked to the changes in mechano-cellular phenotype, which is manifested* via* the variations in tissue’s stiffness, with cancerous tissue stiffer than healthy tissue. Increased tissue stiffness during cancer development may be associated with extracellular matrix protein alignment or overexpression, particularly for different types of collagens, as well as increased matrix fibrosis, cross-linking and vascularization.

In general, AFM has a few promises as a tool for studying the mechanical properties of human tissues. However, when measuring the stiffness map, the precise location on the tissue at where the AFM’s tip makes contact is unknown, leading to an unrevealed relationship between stiffness and local tissue morphology. Because of the small contact area and low indentation of AFM tip, small deformation of the tissue occurs with only small groups of cells. AFM related studies currently cannot be used directly to diagnose all types of bowel cancers. Therefore, a futher advancement is required in AFM technology for the purpose of extracting and testing tissues during biopsy or surgical procedures.^24^

#### Rheological Measurements

Rheological measurement, using a shear rheometer fitted with a parallel plate system, can be used to measure the mechanical properties of tissue samples. Tissues must be first cut into pie-shaped samples with a centimetre-scale punch. In the uncompressed state, the tissue slices range between 3 to 5 mm in height. The fixed lower plate, as shown in Fig. 5b, is used to support and fix the tissue. The upper plate applies a uniaxial compression force to the tissue, followed by a controllable and rotational oscillating force, subjecting the sample to a combination of compressing and shearing force.^24^

In linear tests, the device works by attaching the upper plate to the surface of the tissue, applying constant compression and rotation, and measuring the force using a load cell. A linear position sensor is used to measure the axial displacement of the upper plate. The angle deflection of the upper plate, $\varphi$, can be converted into shear strain $\gamma$* via*

14 $$\begin{aligned} \gamma = \frac{\varphi r}{h}, \end{aligned}$$

where *r* is the radius of the plate, and *h* is the gap height between the rheometer’s plates. The measured torque *M* corresponds to the shear stresses $\tau$ in the sample as

15 $$\begin{aligned} \tau = \frac{2M}{\pi r^3}. \end{aligned}$$

Thus, the basic axial and shear stress–strain parameters can be obtained and analysed to derive Young’s and shear moduli of the tissue.

In Reference 24, two types of rheological tests were carried out during the dynamic shear tests to imitate the periodic deformations expected to occur* in vivo*. The first test involved oscillating shear deformation of the tissue at a constant frequency and with a constant shear amplitude. The second test involved oscillating shear deformation of the sample at a constant frequency with a varying shear amplitude. During oscillating shear deformation in both tests, a uniaxial strain is simultaneously applied in the range of 0–40% of the sample’s initial height. Therefore, the normal stress ($\frac{F(t)}{A_0}$, where *F*(*t*) is the recorded uniaxial force and $A_0$ is the surface area of the upper rheometer plate) and shear stress courses as functions of time were determined. The evolution of shear strain over time can be depicted as

16 $$\begin{aligned} \gamma (t) = \gamma _{\text {a}}\sin (\omega t), \end{aligned}$$

where $\gamma _{\text {a}}$ is the shear strain amplitude and $\omega = 2 \pi f$ is the angular frequency. There is a phase lag in the measured stress in relation to the applied shear strain. The shear stress as a function of time equals

17 $$\begin{aligned} \tau (t)= \tau _{\text {a}}\sin (\omega t + \delta ), \end{aligned}$$

where $\tau _{\text {a}}$ is the shear stress amplitude and $\delta$ is the phase lag between the stress and the strain. Finally, the modulus of elasticity can be calculated from

18 $$\begin{aligned} G^{\prime } = \frac{\tau _{\text {a}}}{\gamma _{\text {a}}}\cos \delta , \end{aligned}$$

which is known as the storage modulus. In addition, the viscous modulus, also called loss modulus, can be expressed as

19 $$\begin{aligned} G^{\prime \prime } = \frac{\tau _{\text {a}}}{\gamma _{\text {a}}}\sin \delta . \end{aligned}$$

Therefore, the $G^{\prime }$ and $G^{\prime \prime }$ were derived as a function of shear deformation under cyclic loading-unloading conditions.

Both the rheological measurement and the AFM indicate a similar conclusion that cancer tissues are much stiffer than the healthy ones, which is reflected either from the Young’s modulus calculated by Eqs. (9)–(11) or the storage modulus obtained by Eq. (18). For the healthy sample, the stress–strain relationship is linear indicating a Young’s modulus of 1.9 kPa, which is consistent with the value obtained by the AFM. For the cancerous tissue, the stress–strain plot is nonlinear, and the local slope of the plot increases with the increasing strain, which is a characteristic of its fibrotic diseased tissues. According to Reference 24, the calculated Young’s modulus of cancer tissues is 18.6 kPa from the initial slope of the strain below 20%, where the tissue response is approximately linear, which is consistent with the values measured by the AFM. Although the rheological measurement has a slightly higher result than the AFM, it is very likely that this is due to the higher strain from the macroscopic measurement and the dominance of the stiffest region of the sample in the macroscopic response. As a result, the main advantage of using shear rheometry is that it can measure the mechanical properties of a large fragment of 3D tissue by providing its averaged mechanical parameters as a whole.

According to the storage modulus results in Reference 24, for 40% tissue compression, the storage modulus of cancer tissues is 9.60 kPa, while that of the normal tissues is 1.52 kPa. Furthermore, the storage moduli of both healthy and cancer tissue increase with the increasing uniaxial strain, but the increase of cancer tissues is nonlinear and faster than that of the healthy ones. On the other hand, the storage modulus decreases with the increasing shear strain for all the healthy and cancerous tissues. It was revealed in Reference 24 through a storage modulus comparison for axial compression and shear strain showing that the tissue could be hardened under axial compression while softened under shear deformation, particularly for the cancer tissue.

#### Tactile Sensor

Tactile sensor is a rigid spherical indenter probe which can accurately detect the vertical variation when it indents into the object. As shown in Fig. 5c, the specimen was placed on a plate with sufficient stability, and the mucosal side was placed upward so that the probe could be directed vertically toward this surface. Measurements of the elastic modulus were performed three times at three different areas of each sample for the normal colon tissues and the bowel cancer tissues in different cancer stages. The elastic modulus of the bowel cancer tissue sample was defined as the mean Young’s modulus of those results.^127^

In Reference 57, tactile sensor was used together with Hertz contact mechanics to determine tissues’ stiffness, and the contact stress theory of Hertz was the same as that used for the AFM. In the test, a rigid spherical probe with a radius of 2.5 mm was indented vertically into the sample for 4.0 mm, and then released at a constant speed. The initial contact pressure was set by a built-in motor. The specimen’s Young’s modulus was calculated for every 5 $\mu$m of indentation depth, and a total loading depth of 1.25 mm was used for analysis. Finally, Poisson’s ratio of the tissue was determined at 0.49.

The resulting median Young’s modulus of bowel cancer tissue was 7.51 kPa for 106 cases, which was significantly higher than the normal bowel tissue at 0.936 kPa. For T-stage evaluation, the median Young’s modulus gradually rises as the stage progresses from T1 to T4. According to Reference 57, T4 had the highest Young’s modulus (13.8 kPa), followed by T1 (2.81 kPa), T2 (5.49 kPa), and T3 (8.89 kPa). Another finding of this work is that an increase in cancer stage is associated with an increase in tumour size. In general, the ‘oldest’ lesions, such as fibrosis and ulcers with extensive exudates on the surface, were found in the tumour’s centre. Tumour’s elasticity is thus related to tumour size and ageing.

#### Ultrasound Elastography

Elastography is a non-invasive medical imaging technique that uses low-frequency vibrations during an ultrasound (US) or magnetic resonance imaging (MRI) to determine the stiffness of organs or the other structures in human body.^122,128^ Particularly, it is useful for detecting the presence and severity of glandular disorders,^85^ myocardial stiffness,^106^ musculoskeletal pathologies,^108^ liver disease,^47^ inflammatory bowel disease^123^ and bowel cancer.^19,29,45,73^ The measurement is to examine how quickly these vibrations move through the organ, and then this information will be used to create a visual map revealing the differences in the elastic properties of soft tissues for further diagnosis. On the other hand, conventional ultrasound diagnostic images can only visualise the differences in soft tissue acoustic properties through the echogenicity of ultrasound waves passing through the soft tissue,^62,68,75^ whereas ultrasound elastography (USE) images (Fig. 5d) are able to reveal the differences in the elastic properties of soft tissues.^117^ Thus, this subsection will focus on the study of bowel cancer by using the USE technique.

The elastic moduli given by Eqs. (5)–(8) are presented in static deformations, but they can also be characteristics of propagation speed of the waves inside the tissue. Wave propagation contains longitudinal waves (compression waves) and transverse waves (shear waves). First, the tissue motion is parallel to the direction of wave propagation, and the speed of longitudinal wave $C_{\text {L}}$ can be defined by the Newton–Laplace equation as

20 $$\begin{aligned} C_{\text {L}}= \sqrt{\frac{K}{\rho }}, \end{aligned}$$

where *K* is the bulk modulus, $\rho$ indicates material’s density. However, relatively small differences in wave speed and *K* for different soft tissues will provide insufficient tissue contrast for elastography measurement.^117,120^ By using the shear modulus *G*, tissue motion is perpendicular to the direction of wave propagation, and the speed of transverse waves $C_{\text {T}}$ is low in soft tissue, allowing suitable comparison in *G* between tissues, which can be expressed as

21 $$\begin{aligned} C_{\text {T}}= \sqrt{\frac{G}{\rho }}. \end{aligned}$$

So, the relationship between Young’s modulus *E*, shear modulus *G* and propagation speed of waves in ultrasound elastography measurement, combining with the formula conversion of Eq. (7), is given as

22 $$\begin{aligned} E = 3G = 3\rho C_{\text {T}}^{2}. \end{aligned}$$

For ordinary ultrasound shear wave imaging, it will directly measure the propagation speed of the wave $C_{\text {T}}$, and convert it to the shear modulus *G* or Young’s modulus *E* by using Eq. (22). However, under high frequency excitation of USE, the role of tissue viscosity cannot be ignored. Therefore, assessing the magnitude of these values will depend on the viscoelastic model of the soft tissue. For example, Deffieux* et al*.^21^ proposed to use the Kelvin–Voigt viscoelastic model to obtain equations for transverse wave velocity, which includes the excitation frequencies.

**Figure 6:**
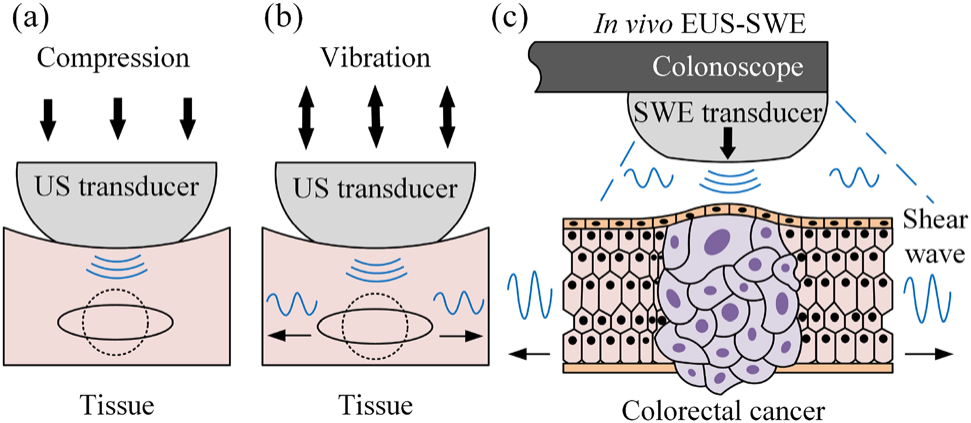
Ultrasound elastography techniques: (a) strain elastography, (b) transient elastography and (c) endoscopic ultrasound shear wave elastography (EUS-SWE). For strain elastography, excitation methods include statically induced displacement through active external compression or passively induced physiological motion to produce normal stress. Dynamic vibration compression at the tissue surface is to produce shear waves in transient elastography. EUS-SWE induced tissue displacement and shear waves by acoustic radiation force impulse excitation have been used in clinical endoscopes for detecting bowel cancer in real time.^26^

In the clinical practice, strain elastography, acoustic radiation force impulse (ARFI) strain elastography, transient elastography and shear wave elastography (SWE) are the main USE techniques for measuring the elasticity of human tissue and can generate corresponding elastograms, which are illustrated in Fig. 6. For the strain elastography, a normal stress is applied by the operator’s regular compression at the tissue’s area of interest, and the normal strain is measured* via* ultrasound echo time as presented in Fig. 6a. Then the elastogram (strain distribution contour map) will be obtained and displayed on the external monitor device and such practice can be found in the colonic diseases practice, e.g., Reference 32. Here, Eq. (5) can be used to provide a qualitative evaluation of Young’s modulus. However, the magnitude and depth of the applied force at each time may affect the reference standard of the results when comparing different areas.^108^ An acoustic pushing pulse that has the ability of short-duration and high-intensity is used by the ARFI strain elastography to replace hand compression for tissue displacement in the normal direction.^120^ For transient elastography, as a shear wave imaging technique,^120^ a controlled mechanical vibration on the ultrasound transducer is used to impact the tissue vertically to generate shear waves inside the tissue that are perpendicular to the impact direction, see Fig. 6b. Then the average shear wave speed is measured and converted to Young’s modulus by using Eq. (22).

For shear wave imaging, transient elastography needs to be in contact with the tissue and provides mechanical strike, thus bringing limitations to the measurement, especially for the complex structure of the tissue and the location of the organ that are far away from the source of excitation. The newest shear wave imaging is to use ARFI technique to generate shear waves. Unlike the ARFI strain elastography, the acoustic pushing pulse produces longitudinal waves first, and then converts to shear waves inside the SWE transducer, which is shown in Fig. 5d. For *in vivo* gastrointestinal tract examination, endoscopic ultrasound shear wave elastography (EUS-SWE), as illustrated in Fig. 6c, which uses a colonoscope equipped with an onboard SWE transducer has been used for clinical diagnosis. Shear waves can propagate in a near-cylindrical conical space, allowing real-time monitoring of shear wave velocities and Young’s modulus, and generating quantitative elastic elastograms.^28^ Chen* et al*.^19^ stated that cancer’s stiffness is the best staging criterion among all features to assess the staging of rectal tumours. Young’s modulus of rectal adenoma measured by SWE is 14.8 ± 9.5 kPa. Different stages of the cancers may increase gradually, e.g., T1, T2 and T3 stages are 54.7 ± 11.4, 87.3 ± 20.3 and 157.3 ± 41.0 kPa, respectively, whereas the stiffness of normal rectal wall is 9.9 ± 3.0 kPa. By using the same technique, Fan* et al*.^29^ identified slightly lower stiffness but with a similar tendency on Young’s moduli for different cancer stages, which T1, T2 and T3–T4 stages are 29.6 ± 11.2, 76.7 ± 33.1 and 104.8 ± 20.5 kPa, respectively. Deptuła* et al*.^24^ argued that despite ultrasound has undeniable advantages, SWE methods have drawbacks due to the complex structure of the tissue and the location of the organ which is far away from the source of excitation. The results strongly depend on the excitation frequency, and this is often greater than the time scale that cells can respond to. Nevertheless, EUS-SWE is still the most effective means to diagnose bowel cancer and its biomechanical properties in real-time with the advent of artificial intelligence (AI) in future clinical practices.

#### Summary and Remarks

Table 1 summarises the Young’s moduli of normal and cancerous bowel tissues measured by different methods reviewed above. The increase of cancerous tissue’s stiffness with cancer stage was manifested strongly in all these measurement methods. As the cancer cells develop and become more invasive, the size of cancer tissue increases as well. Mechanical properties of local tissue surface can be measured by AFM, whereas rheological measurement, tactile sensor and ultrasound can reflect the averaged properties of a large area of the tissue surface, which are valuable for investigating the biomechanical properties of the whole tumour.

**Table 1:** Summary of stiffness values of bowel cancer tissue in different investigation methods.

Method	Young’s modulus (kPa)			
	Normal tissue	Tumour stage	Cancer tissue	References
AFM	0.44 ± 0.3	–	5.80 ± 3.8	24
Rheological measurement	1.9	–	18.6	24
Tactile sensor	0.936 (0.374–7.33)	T1	2.81 (1.91–3.96)	57
		T2	5.49 (1.08–12.2)	
		T3	8.89 (2.60–43.2)	
		T4	13.8 (5.58–68.0)	
		T1–T4	7.51 (1.08–68.0)	
SWE	8.4 ± 3.6 (2.7–16.4)	T1	29.6 ± 11.2 (10.9–52.2)	29
		T2	76.7 ± 33.1 (23.9–126.3)	
		T3–T4	104.8 ± 20.5 (65.1–137.1)	
SWE	9.9 ± 3.0 (6.2–17.0)	Adenoma	14.8 ± 9.5 (8.8–26.9)	19
		T1	54.7 ± 11.4 (40.9–70.3)	
		T2	87.3 ± 20.3 (56.4–112.0)	
		T3	157.3 ± 41.0 (70.8–277.2)	

The Young’s moduli under the method of tactile sensor are the medians. The rest data are means or means ± standard deviation. Data ranges are from the minimum to the maximum in parentheses

The observed changes in the mechanical response of cancer tissues may allow the identification of specific mechanical markers. In particular, stiffness could be a new mechanomarker for bowel cancer and potentially for the other bowel pathologies as well. In the future, assessment of Young’s modulus of bowel cancer during colonoscopy or capsule endoscopy may enable physicians to accurately evaluate a patient’s cancer stage and metastasis before surgery.

## Existing Diagnostic Techniques

Various methods currently exist for bowel cancer treatment and these include removal of affected tissues* via* surgery, targeted killing of affected cells using chemotherapy and radiotherapy, and also cancerous cells growth retardation* via* metastasis prevention therapies.^90^ However, proper diagnosis must be carried out before any treatment can be offered. Between 2013 and 2017, England cancer treatment data showed that patients have 98, 93, 89 and 44% survival rate within a year of treatment, if diagnosed at T1, T2, T3 and T4 stages, respectively.^5^ This thus makes early and accurate diagnosis of bowel cancers of crucial importance to their treatment. In the UK, bowel cancer screening is offered every two years to patients over 60 to 74 years even if without symptoms, with a future plan to reduce it to 50 years.^87^ In the US it is offered every five years to adults above 50 years and with increased risk, and every 10 years for those without additional risks.^144^ The high mortality rate of bowel cancer at this present-age of scientific and technological advancement suggests that investigating new and advanced screening techniques is an important research goal. Over the years, quite a number of studies have been carried out on bowel cancer detection using different techniques, but only a few have been successfully commercialised. Research is ongoing to improve on presently used diagnostic methods. Some of the major clinical bowel cancer diagnostic methods which are currently in use are briefly reviewed in this section.

### Observable Clinical Symptoms

Like every other disease, bowel cancers also cause observable symptoms and clinical syndromes, and these include changes in bowel habit, rectal bleeding, anaemia (iron-deficiency), weight loss, intestinal obstruction, abdominal pain, and intestinal perforation.^136^ These symptoms can vary depending on the location of the cancer, that is right or left side of the colon.^131,136^ The average age at presentation of right-sided cancers is slightly lower than that for left-sided cancers. It should be noted that, with most bowel cancer patients being asymptomatic at the early stages, and some symptoms, such as rectal bleeding, being non-specific and associated with other common diagnoses (such as anal fissure and haemorrhoids), additional measures are required for proper diagnosis, including physical examination and investigations.

At their late stages, both right- and left-sided cancers may present a palpable mass within the abdomen or rectum during physical examination. Digital rectal examination involves doctors feeling the inside of the rectum using their fingers. Before this step, a faecal immunochemical test might have also been carried out to test for tiny traces of blood in faeces. The efficacy of individual digital rectal exams and faecal immunochemical tests (FIT) in preventing mortality from bowel cancer is yet unknown but their modest effect cannot be discarded.^40,97^ FIT is often the first step utilised in bowel cancer screening programmes,^14,91,110^ and abnormal results often lead to patients being referred for further tests, including colonoscopy or flexible sigmoidoscopy.

### Laboratory Tests

Complete (or full) blood count is a common laboratory test often carried out by doctors to assist with the evaluation of the current risk of bowel cancer. It measures the different types of cells in the blood and studies have shown patients with bowel cancers to be deficient of red blood cells, haemoglobin and mean corpuscular volume as a result of the unseen bleeding of cancerous polyps over an extended period of time.^125,139^ Mellor^82^ has shown patients diagnosed with bowel cancer to have higher levels of red blood cell distribution width, white blood cell count, and platelets compared to undiagnosed patients. Blood may also be tested for carcinoembryonic antigen, which is a marker chemical substance sometimes released by bowel cancer tissues. It is a glycoprotein present in normal mucosal cells but occurs in higher amounts in adenocarcinoma tissues, such as colorectal cancer tissues. Sensitivity and specificity are low, so this test is often used for monitoring during and after treatment rather than for screening or diagnosis.^135^ Tracked monitoring of carcinoembryonic antigen level in a patient’s blood sample can be useful for understanding the prognosis and response to treatment.^11,70^ Complete blood count is often used alongside other tests as its levels can be normal in patients with bowel cancer or abnormal in many other conditions. Coupled with other tests and observable patient factors, it has also been integrated with machine learning in efforts to improve early colorectal cancer detection, see e.g., Reference 43.

Another laboratory test usually conducted is biopsy, which involves the extraction of a small sample of cells and tissue from the cancerous section of the bowel during colonoscopy or surgery, the sample is then subjected to various analyses under a microscope. Alongside the usual analyses, different research groups have tested the mechanobiological properties of extracted tissues. Their results have shown that living cells tend to possess different mechanobiological properties, including viscosity and stiffness, when affected by diseases like cancer, and variations in these properties have been proposed as biomarkers for early-stage detection.^10,103^

### Endoscopies

Endoscopy is a medical procedure that is used for screening the internal parts of the digestive tract using an inserted or a navigated instrument.^15^ Procedures using inserted instruments include colonoscopy and sigmoidoscopy. Colonoscopy attempts to examine the entire internal lining (mucosa) of the large bowel, whilst sigmoidoscopy examines just the lower part, using a flexible tube with lights and a camera at its end. The tube is passed into the bowel through the anus and it is gently negotiated through the colon. Images are then viewed on a monitor to allow the detection of visible abnormalities. With these methods, advanced stage cancer lesions are easily detected by the endoscopist, while early stage cancers are quite difficult and often appear as subtle mucosal lesions.^22^ To improve detection, optimal bowel cleansing is required before examination, and mucosa is carefully and painstakingly inspected over many minutes. As at 2019, around 900,000 colonoscopies were reported to be carried out each year in the UK, and this is expected to rise to over a million in future years.^74^

With this increasing demand, the associated discomfort,^58^ the risk of infection^66^ and the intense training requirement^119^ of colonoscopy, alternative and less invasive endoscopies have been developed. An example of such is the capsule endoscopy also referred to as video capsule endoscopy. It involves the use of a pill-sized video camera to examine the internal lining of the bowel.^12,60^ The patient swallows the capsule, which contains a small disposable camera that takes series of images as it travels through the digestive tract. The pictures are either stored on-board or are wirelessly transmitted to a worn external recorder. Most capsules (including all those designed for colonic investigation) are propelled by peristalsis,^48^ taking about 7 to 10 h to traverse the entire gastrointestinal tract, while some are propelled by interaction between an embedded magnet and external magnetic fields from coils or permanent magnets.^146^ Another propelling mechanism which has not been used in current clinical prototypes is the embedded actuators with locomotive legs,^109^ and it is sometimes combined with magnetic fields to form a hybrid prototype of capsule.^121^ A design drawback of the last two capsule types is that coupling actuators with independently moving legs require a power source that may be difficult to fit into an ingestible capsule. In addition, the independently moving legs could potentially injure the intestinal wall.

An on-going development is the self-propelled vibro-impact capsule^71,72^ whose locomotion is self-propelled and controllable. It is capable of both forward or reversed steering for the purpose of reviewing and verification of suspicious lesions, whilst also reducing procedural time.

Since their introduction,^48,129^ endoscopic capsules have been a major focus of recent endoscopy research, much of which relates to their propulsion through the gastrointestinal (GI) tract. Amongst the current clinical and commercially available capsules, majority of which are propelled by the peristaltic contraction^64^ of the GI muscles, include PillCam from Medtronic,^80^ Capsocam plus from CapsoVision,^16^ C-Scan cap from check-cap^18^ and the Capsules Endoscopy Systems from Jimhans medical.^52^ Others are propelled using the interaction between internally embedded magnets and external magnetic fields from permanent magnets or magnetic coils, and are mainly used to examine the stomach rather the intestines.^146^ These include the EndoCapsule from Olympus,^99^ OMOM robotic capsule from Jinshan Group,^54^ Mirocam from IntroMedics,^49^ Navicam from AnX Robotica,^2^ and Dasheng Capsule endoscopic system from JIFU Medical.^51^ Magnetically-propelled capsules offer potentially better flexibility and faster transit times, but their performance may be reduced in those with body mass index (BMI) greater than 38,^2^ as the interaction between the internal and external magnetism tends to be weakened as BMI increases. It is unknown whether the frictional forces applied to the intestinal wall when using such magnetic propulsion are potentially injurious.

At completion, endoscopic capsules are passed out through the anus and the collated images are analysed by a clinician for diagnosis. Being a relatively new method, colonic capsule endoscopy^153^ is often proposed as a clinical filter for patients before referral for colonoscopy and has been found to reduce the need for colonoscopy by 71%.^42^ Compared to normal colonoscopy, capsule endoscopy has been found to cause less patient discomfort with improving detection of significant-sized polyps of > 9 mm, thus potentially increasing patients’ participation in screening programmes.^35,61,134^

Due to the large volume of images often resulting from endoscopy, most endoscopy systems are being integrated with artificial intelligence (AI) to assist with interpretation. These are referred to as computer-aided detection (CADe) and computer-aided diagnosis (CADx) systems. The CADe systems are useful for locating lesions in the medical endoscopy images while the CADx systems are useful for characterising the lesions, such as differentiating between benign and malignant lesions. Examples of currently used AI models include Endoscreener from Wision A.I.,^141,145^ GI Genius from Medtronic,^38,81,111^ ENDO-AID from Olympus,^67^ CAD EYE from Fujifilm,^31^ Discovery from Pentax^101^ and EndoBRAIN from Cybernet.^63,84^

In the last few years, robotics has increasingly played significant roles in medicine both in disease diagnosis and treatment. For endoscopy procedures, an attempt to further improve patient experience while also enabling easier navigation and visualisation of the complex GI system has led researchers to further explore a variety of robotic engineering systems. Some of the explored systems have led to the invention of robotic endoscopy systems that differ from each other based on their locomotion strategy. Examples include pneumatically driven soft snail robot systems that mimic snail locomotion,^17,147,148^ electromagnetically driven soft-tethered colonoscope^69,78^ and the earthworm-like soft robot inspired by earthworm locomotion.^41,142,143^ Most of these robotic endoscopy systems are however yet to make it into the commercial market. The earlier mentioned magnetic,^146^ vibration^72^ and locomotive legs^109^ propelled capsule systems belong to this class of robotic endoscopy. Aside the invention of robotic endoscopy systems, the study of robotic engineering systems and artificial intelligence has also led to the development of autonomous control system for endoscopy procedures.^46,78,149^ Compared to the manually controlled endoscopes, autonomous endoscopes have been found to be more effective in terms of improved patient safety, shorter travel time and higher diagnostic rate, see e.g., References 46, 78, and 153.

### Computed Tomography Colonography

Computed tomography (CT) colonography which is also referred to as virtual colonoscopy is another less invasive modality used increasingly in bowel cancer diagnosis. It examines the bowel using 2D and 3D imageries reconstructed from a CT scan.^8,88^ CT scans use X-ray imaging to produce a sliced through image of body parts. Recent advancements in computing power have permitted CT colonography to be carried out much more quickly than before, both improving the quality of imaging and reducing patients’ exposure to radiation, whilst acquiring sufficient data for accurate polyp and lesion detection. The method is minimally invasive and it is often recommended for patients with medical conditions that render other methods unsuitable. Like regular colonoscopy, patients may be asked to undergo bowel cleaning before the procedure or, alternatively, they may have to ingest an iodine-based contrast agent with meals two days before the procedure. During the procedure, the bowel is inflated using air or carbon dioxide gas from a narrow tube passed into the patient's rectum. To enable the radiographer to capture clear scans of the bowel, the scans are carried out with the patients lying on their back and front while holding their breath for about 20 s in both positions.^9^ Studies have shown the effectiveness of CT colonography to improve with increasing lesion sizes. Earlier studies demonstrated sensitivities of around 75–80%.

### Magnetic Resonance Imaging

MRI uses strong magnetic and radio waves to produce an inside picture of the bowel. It gives a more detailed view of the planes between different tissues compared to a CT scan, hence, it is often used preoperatively in rectal cancers to deduce if all the cancers can be surgically removed or if radiotherapy will be used first.^156^ However, it is never recommended for patients with heart monitors, pacemakers or other metallic surgical clips as these are prone to defects or may cause injury due to the magnets in the scanner. Nerad* et al*.^92^ investigated the use of MRI for bowel cancer staging in 55 patients with biopsy-proven bowel cancer. They reported high sensitivity (72–91%) for invasions through bowel walls and low sensitivity (43–67%) for invasion beyond bowel wall of $\ge$ 5 mm or invasion of surrounding organs. Pang* et al*.^105^ investigated the potential of a magnetic resonance image tumour-regression grading (MRI-TRG) system to predict pathological TRG during the treatment of a locally advanced rectal cancer using neoadjuvant radiochemotherapy. The MRI-TRG displayed sensitivity as high as 90.1% and specificity of 92.8%. MRI scans have also found usefulness in assessing and predicting patient response to bowel cancer treatment.^7,124^

### Positron Emission Tomography Scan

A positron emission tomography (PET) scan makes use of a small injected dose of a radio-labelled sugar, such as fluorodeoxyglucose-18, as a tracer to monitor the metabolic activities of cells in different parts of the bowel. Most cells utilise glucose in their metabolism, and cancerous cells tend to have high metabolic activity and thus absorb more sugar than healthy cells. PET scans are mostly either combined with CT scans (PET-CT scan)^76,113,151^ or MRI scans (PET-MRI scan)^113,137^ to produce a detailed localisation of areas of high metabolic activity. These areas are analysed to find out if they represent cancerous (malignant) or non-cancerous (benign) processes. As well as delineating primary tumours within the large bowel, PET scans are particularly useful for detecting metastases, assessing the progress of an ongoing treatment, pointing out areas for potential biopsy and to plan radiation therapy. Recent studies have focused on the use of artificial intelligence in PET image processing and interpretation.^30,56^

## Conclusions and Future Outlook

In this review, we have outlined the complex and multifactorial nature of bowel cancer, the aetiology of which includes endogenous (e.g. genetic) and exogenous (e.g. microenvironmental changes and lifestyle) components. Epidemiologically, bowel cancer is described as sporadic, familial or hereditary. However, most bowel cancer cases are sporadic and caused by environmental factors rather than genetic predisposition or family history. In terms of pathogenesis, most colon cancers are thought to develop directly from adenomatous polyps. The adenoma-carcinoma sequence links the sequence of genetic alterations and tumour morphogenesis to different stages of tumour development, from promoting benign tumour formation to the final step that transforms the tumour into invasive carcinoma. Once a tumour invades the submucosa, it can penetrate the lining of the colon and invade nearby structures, the depth of invasion corresponding to different cancer stages. The genomic instability of bowel cancer and its pathogenesis are mediated by three major pathways: chromosomal instability, microsatellite instability, and CpG island methylation phenotypes. In addition, changes in the gut microenvironment caused by variations and alterations in the gut microbiome are one of the mechanisms proposed to link environmental factors to mutagenesis and other genomic changes.

We have also discussed the stages in the process of bowel cancer metastasis. Current consensus in the literature suggests that environmental factors local to the primary tumour in the colon contribute to the initial stages of the metastatic cascade including angiogenesis, invasion and intravasation. The circulatory system is the primary route for the dissemination of CTCs to the rest of the body and mechanical changes in cell structure could aid CTCs in resisting destructive shear forces in the blood. Extravasation is facilitated by the binding of CTCs to blood vessel walls or by physical entrapment in the small diameters of capillaries. In the final stage of the metastatic cascade, the extravasated tumour cells first form micrometastatic tumours, and then macrometastatic tumours in the new metastatic site, usually the liver. However, more clinical studies could improve the current understanding of the mechanisms that contribute to metastasis in bowel cancer.

In recent decades, the biomechanical properties of bowel cancer have emerged as potentially useful biomarkers that might improve our understanding of the physiological and pathological processes at the cell, extracellular matrix and tissue levels, as well as providing design guidelines for future functional diagnostic devices (e.g. endoscopes and capsule robots). In order to provide a rudimentary understanding of the mechanical properties of healthy and tumour tissues and the potential cancerous transition, different existing detection methods and relevant findings combined with their corresponding pathological phenomena were summarised, including methods such as atomic force microscopy, shear rheometer, tactile sensor and ultrasound elastography. Although different measuring approaches reveal differentiated results in cancer tissues, the increase in cancerous tissue’s stiffness with cancer stage was manifested strongly in all these measurement methods, with the mean values of Young’s moduli for normal bowel tissues falling significantly below those for all bowel cancer stages. In addition, lesions that experienced pathological changes a long time ago are larger and display central sclerosis and fibrosis, demonstrating that a tumour’s elasticity will relate to its age and size. Finally, it is suggested that different methods of measuring tissue stiffness could be referenced and incorporated into the future design of functional real-time devices, perhaps incorporating artificial intelligence, in order to both detect cancers and polyps and assess tumour stage* in vivo*. A device that can assess these parameters at the core of a mass lesion may prove the most useful addition to the current diagnostic armamentarium.

In addition, we have summarised briefly above those techniques currently used in clinical practice to diagnose and stage bowel cancer. These include ordinarily observable symptoms, simple laboratory tests on blood samples, faeces or extracted tissues, and complex medical examinations such as colonoscopy, capsule endoscopy, CT colonography and PET scans. Such procedures may be combined to improve diagnostic performance, whilst some may be used only as a clinical filter before other tests are prescribed. A major drawback on the use of many of these techniques is their reliance on visual and post-developmental features such as numbers, sizes and shapes, which renders them less sensitive and specific for the detection of early and hard-to-visualise bowel cancers and precursor lesions. With bowel cancer survival being so dependent on stage at diagnosis, much research is now focused on non-visual, early-stage, diagnostic methods. This is based on the fact that the earliest signs of cancer development are changes in intrinsic tissue properties rather than visible observations. The success of these lines of research could be a milestone in the global aim of reducing bowel cancer mortality and extending patients’ survival.

In summary, it is clear that, despite improvements in bowel cancer screening, detection, staging and treatment over recent years, there remains much work to do to meet the global aim of dramatically reducing both the mortality and suffering caused by bowel cancer, and the burden of the disease on patients, families, health systems and societies. The health care community and society in general aspire for the development of new technologies that can simply, cheaply, reliably and non-invasively detect cancer and its precursor lesions at the earliest possible stage, when the chance of cure is high and when treatments may be less morbid and toxic. Furthermore, the accurate determination of tumour stage, including the presence of CTCs and micro-metastases, during this assessment could enable clinicians and patients to develop truly individualised treatment plans with the highest chance of cure. It is hoped that a greater understanding of the biomechanics of bowel cancer, within the colon, within the circulation and at sites of metastasis, is one avenue of research with the potential of meeting some of these aspirations.
